# On the performance of pre-microRNA detection algorithms

**DOI:** 10.1038/s41467-017-00403-z

**Published:** 2017-08-24

**Authors:** Müşerref Duygu Saçar Demirci, Jan Baumbach, Jens Allmer

**Affiliations:** 1 Molecular Biology and Genetics, Izmir Institute of Technology, Urla, Izmir 35430 Turkey; 2 Computational Systems Biology, Max Planck Institute for Informatics, 66123 Saarbrücken, Germany; 3 Computational Biology, University of Southern Denmark, DK-5230 Odense M, Denmark; 4 Bionia Incorporated, IZTEKGEB A8, Urla, Izmir 35430 Turkey

## Abstract

MicroRNAs are crucial for post-transcriptional gene regulation, and their dysregulation has been associated with diseases like cancer and, therefore, their analysis has become popular. The experimental discovery of miRNAs is cumbersome and, thus, many computational tools have been proposed. Here we assess 13 ab initio pre-miRNA detection approaches using all relevant, published, and novel data sets while judging algorithm performance based on ten intrinsic performance measures. We present an extensible framework, izMiR, which allows for the unbiased comparison of existing algorithms, adding new ones, and combining multiple approaches into ensemble methods. In an exhaustive attempt, we condense the results of millions of computations and show that no method is clearly superior; however, we provide a guideline for biomedical researchers to select a tool. Finally, we demonstrate that combining all of the methods into one ensemble approach, for the first time, allows reliable purely computational pre-miRNA detection in large eukaryotic genomes.

## Abstract (web summary)

As the experimental discovery of microRNAs (miRNAs) is cumbersome, computational tools have been developed for the prediction of pre-miRNAs. Here the authors develop a framework to assess the performance of existing and novel pre-miRNA prediction tools and provide guidelines for selecting an appropriate approach for a given data set.

## **I**ntroduction

Since their discovery, about two decades ago, microRNAs (miRNAs) have been detected in a large number of organisms including microbes^1^, sponges^2^, metazoan^3^, plants^4^, and viruses^5^. Nowadays, miRNAs are considered important factors in many human diseases and they are believed to be good candidates for disease markers and therapeutics^6^. In plant breeding studies miRNAs are applied to control agronomical traits such as tolerance to biotic and abiotic stress factors, to increase yield, modify fruit development, and influence crop quality^7, 8^.

Dysregulation of miRNAs is a hallmark of diseases; among them cancer^9^, that makes miRNAs interesting as biomarkers^10^, even more so since they are often detectable in bodily fluids^11^ and thus are accessible with low-invasive methods. Modulation of miRNA abundances may lead to therapeutics^12^ that may prove to be especially useful in personalized medicine^13^. Particularly when taking into account miRNAs’ possible roles in cell-cell (circulating miRNAs)^14^ and pathogen-host communication^15^. Experimental detection of miRNAs can be achieved using miRNA profiling approaches, such as microarray, quantitative real-time PCR (qPCR), and deep sequencing technologies^16–18^. There exist some challenges in employing such experimental methods^19^. For instance, qPCR-based and microarray miRNA experiments suffer from low specificity and need extensive normalization^16, 18^. Both approaches also cannot detect novel miRNAs^19^ since either primer (qPCR) or target sequences (microarray) need to be pre-determined. This need for a priori knowledge does not pose a problem for sequencing-based approaches, but they are hampered by the need for extensive downstream computational analyses, such as by tools covered and discussed here^19^. Finally, a miRNA’s effect is in the best case established on the protein level, and therefore all approaches need to be amended with Western blot or mass spectrometry analyses, adding significantly to the experimental complexity^20^.

Considering the massive impact of miRNAs especially in human disease and plant breeding (i.e., human nutrition) and taking into account our inability to experimentally determine all pre-miRNAs, it is crucial for the community to be able to rely on computational methods for pre-miRNA detection.

Although many tools for the detection of pre-miRNAs have been developed (Table 1) a number of key issues remain unaddressed. The first problem is that many of the tools do not provide a readily working implementation (Table 1), which makes it impossible for researchers to select the right tool for their data. Moreover, the most popular tool according to Google Scholar citations will turn out to be, in general, the least effective one according to our findings (see below). It is thus evident that guidelines for tool selection need to be provided to enable researchers to make an informed choice when selecting a tool. In addition, performance comparison among tools cannot be done based on their published metrics since they are based on different data sets, used different approaches to establishing pre-miRNA detection models, and present diverse performance measures. A unified, unbiased evaluation was lacking. Lastly, previous evaluations have been performed for a subset of the state of the art, but only when a new tool was to be published and using different data as well as varying parameters, which obfuscates comparative evaluations.

**Table 1:** Available pre-miRNA detection tools

Study	ML algorithm	Feature number	Positive data	Negative data	Sampling	Implementation	Number of citations (Google Scholar)
Xue^46^	SVM	32	MiRBase 5.0	CODING dataset (Pseudo)	Random selection (approx. 1:1 positive negative ratio)	*	412 (34)
Jiang^47^	RF, SVM	34	MiRBase 8.2	pseudo	Random sampling (approx. 1:1 positive negative and 1:1.5 training testing ratio)	*	376 (48)
Ng^37^	SVM	29	MiRBase 8.2	pseudo	Random selection without replacement (1:2 positive negative ratio)	*	203 (19)
Batuwita^48^	SVM	21	MiRBase 12	pseudo & Human other ncRNAs	Outer-5-fold-cv	+	172 (16)
Xu^49^	A novel ranking algorithm based on random walks & SVM	35	MiRBase (September 1, 2007)	Random, non-overlapping 90nt fragments from the human genome	Random selection (1:2 positive to negative ratio)	*	80 (4)
Ding^50^	SVM	32	Known miRNAs	UTRdb & ncRNA from Rfam 9.1	Outer 3-fold cross-validation	−	61 (11)
Chen^41^	LibSVM	99	miRBase (2013)	pseudo & Zou	Leave-one-out	+	31 (24)
Burgt^51^	*L* score classifier	18	non-plant miRNA hairpin sequences (miRBase version 9.0)	–	10-fold cross-validation	*	31 (4)
Gudys^40^	NB, MLP, SVM, RF, APLSC	28	MiRBase 17	From genomes and mRNAs of ten animal and seven plant species as well as 29 viruses	Stratified 10-fold CV	+	27 (5)
Ritchie^52^	SVM	36	Murine miRBase v17	Transcripts without evidence of processing by Dicer	–	−	20 (5)
Bentwich^53^	–	26	Hairpins from Human Genome	10000 hairpins found in non-coding regions		−	20 (2)
Lopes^54^	SVM, RF, G^2^ DE	13	MiRBase 19	pseudo	Non-standard training and testing scheme.	*	16 (6)
Gao^55^	SVM	57	MiRBase v20	Exonic regions of our some available genomes and ncRNAs from rFam	1:1 positive to negative ratio	*	11 (1)

*SVM* support vector machine, *NB* Naïve Bayes, *MLP* Multi-Layered Perceptron, *RF* Random Forest, *APLSC* Asymmetric Partial Least Squares Classification, *G2DE* Generalized Gaussian Density Estimator, *+* implementation exists, *−* no implementation, * experienced problems with the implementation

Previously published studies performing ab initio pre-miRNA detection using machine learning (*ML*). Listed are the number of features that were effectively used, the training data that was employed and whether an implementation is available

The negative data (see Online Methods) “pseudo” was generated by Xue^16^ but downloaded from Ng^17^. The Table is sorted by the number of citations in Google Scholar (please note that there is a relationship between year of publication and number of citations, therefore, the number of citations in 2016 is provided in parentheses, as well)

Here, we introduce the first neutral, comprehensive, and quantitative evaluation of the state of the art in ab initio pre-miRNA detection. To cope with the combinatorial complexity, we developed izMiR, a freely available platform (http://jlab.iyte.edu.tr/software/izmir), which allowed the exhaustive application of the 13 tools compared in this study. The izMiR framework further enables the streamlined developing of new approaches and repurposing of the methods provided for specific scenarios for the community.

In the following, we present an in-depth comparison of the 13 most popular tools (Table 1). We employed all published positive and negative data sets and added eight additional ones for tool comparison (Table 2). Part of the data was used in training and testing, and those data sets provide intrinsic, whereas, remaining data sets provide extrinsic performance measures. For both kinds, we record ten statistics, but only discuss accuracy and the area under the receiver operator characteristic (area under the curve AUC) in the following although each of the measures could be used for the ranking of the 13 approaches. Using our izMiR framework, for each of the 13 tools, we picked the best models from 3000 trials to represent it for further analysis. These models were applied to all known pre-miRNAs and to thousands of sequences that likely host no miRNAs leading to millions of calculations. All izMiR results are stored within the framework and can be directly inspected at every step of the calculation. Thus, integration and comparison with future results is seamlessly supported without the need for computationally expensive recalculations.

**Table 2:** Data sets

Dataset	Type	Size	Property	Source
hsa	Positive	1881	All human miRNAs in miRBase	http://www.mirbase.org
mirbase	Positive	28596	All miRNAs available in miRBase	http://www.mirbase.org
mmu	Positive	1193	All mouse miRNAs in miRBase	http://www.mirbase.org
mmu*	Positive	380	Mouse miRNAs in miRBase (RPM > = 100)	http://www.mirbase.org
mirgenedb	Positive	1434	All miRNAs available in MirGeneDB	http://www.mirgenedb.org
hsa+	Positive	523	All human miRNAs available in MirGeneDB	http://www.mirgenedb.org
mmu+	Positive	395	All mouse miRNAs available in MirGeneDB	http://www.mirgenedb.org
gga+	Positive	229	All chicken miRNAs available in MirGeneDB	http://www.mirgenedb.org
dre+	Positive	287	All zebra fish miRNAs available in MirGeneDB	http://www.mirgenedb.org
NegHsa	Negative	68046	Extracted from genome and mRNAs of H. sapiens	http://adaa.polsl.pl/agudys/huntmi/huntmi.htm
Zou	Negative	14246	Extracted from coding regions	http://datamining.xmu.edu.cn/main/~leyiwei/mirnaDetect.html
pseudo	Negative	8492	Popular, used in many studies, constructed by using the protein coding sequences (CDSs) of human RefSeq genes with no known alternative splice events	http://web.bii.a-star.edu.sg/archive/stanley/Publications/Supp_materials/06-002-supp.html
Chen	Negative	3054	Excerpt of the combination of Zou and Pseudo	http://bioinformatics.hitsz.edu.cn/iMiRNA-SSF/Material.jsp
NotBestFold	Negative	1881	Created by not using the best fold proposed by RNAFold for human hairpins from miRBase	http://jlab.iyte.edu.tr/software/izmir
Shuffled	Negative	1423	Created by shuffling hsa data	http://jlab.iyte.edu.tr/software/izmir
hsa_FR_	Positive	5000	Created by random number generation between minimum and maximum for all features (hsa)	http://jlab.iyte.edu.tr/software/izmir
hsa_BQ_	Positive	5000	Created by random number generation between lower and upper quartile for all features (hsa)	http://jlab.iyte.edu.tr/software/izmir
hsa_AM_	Positive	5000	Created by random number generation between 40th and 60th percentile for all features (hsa)	http://jlab.iyte.edu.tr/software/izmir
pseudo_FR_	Negative	5000	Created by random number generation between minimum and maximum for all features (pseudo)	http://jlab.iyte.edu.tr/software/izmir
pseudo_BG_	Negative	5000	Created by random number generation between lower and upper quartile for all features (pseudo)	http://jlab.iyte.edu.tr/software/izmir
pseudo_AM_	Negative	5000	Created by random number generation between 40th and 60th percentile for all features (pseudo)	http://jlab.iyte.edu.tr/software/izmir

List of positive and negative data sets used to create and evaluate pre-miRNA detection tools. The first 13 rows refer to previously available data sets whereas the latter 8 are created for this study

We condense the final results of more than 20 million computations into a final summary and guideline. First, we observe that no tool significantly outperforms all other tools on all data sets. We, therefore, consider ensemble methods which unify all 13 tools into 6 different predictors. These ensemble predictors, when analyzed in the same manner as the single tools, provide a significant boost in prediction performance. In general, the Average_DT_ ensemble classifier works best.

## Results

### Comparison of available tools

**Fig. 1:**
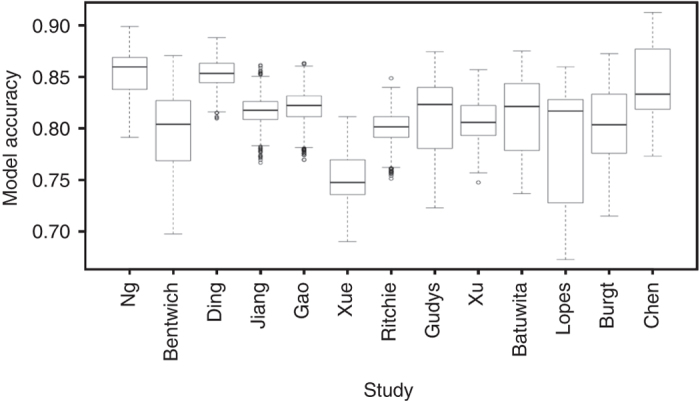
Classifier accuracy distribution. *Box-whisker plots* showing the accuracy distribution among selected studies for 1000-fold MCCV. The individual accuracy measures of the DT, NB, and SVM classifiers were merged to create this plot. Per classifier results can be found in Supplementary Figs. 1–3

Uniform implementations for all tools evaluated in this study were created since few of the original tools have been available and functional (Table 1). For our analysis, we used three machine learning algorithms, decision trees (DT), support vector machines (SVM), and naive Bayes classifiers (NB) (Online Methodology; Fig. 1). Figure 1 provides an overview of the accuracy distribution using 1000 fold Monte Carlo cross-validation (MCCV)^21^ for the averaged performance of all three classifiers (per classifier distributions are also available: DT: Supplementary Fig. 1, NB: Supplementary Fig. 2, and SVM: Supplementary Fig. 3; Supplementary Table 1).

It can be directly observed from Fig. 1 that there is no universally best model and this is further supported by Supplementary Figs. 1–3 and Supplementary Tables 2, 3.

Figure 1 indicates that although Chen_avg_ has the highest absolute performance, Ng_avg_ and Ding_avg_ displayed better overall performance since their accuracy distribution is much less data- and/or classifier-dependent (Supplementary Tables 2, 3) than Chen_avg_, and since the difference among their maximum accuracy is not very large (Chen_SVM_: 0.91, Ng_DT_: 0.90, and Ding_NB_: 0.89). The results further show that all models perform much better than random guessing (0.5); but also that none significantly outperforms all others. Therefore, we designed six consensus models integrating the best models from all studies (refer to “Methods” section and http://www.nature.com/protocolexchange/protocols/4919). These consensus models were compared to the individual studies regarding their receiver operator characteristic curves (Supplementary Figs. 4, 5). The Average_DT_ model performed best reaching an AUC of 0.99, thereby being much better than the next best models Ding_Dt_ (AUC: 0.93) or Chen_DT_ (AUC: 0.91).

### Model performances

The performance of the generated models is data dependent and, therefore, we applied the NB and the DT models to all published positive and negative data sets as well as to eight novel ones (Table 2). Positive data sets derive from miRBase and MirGeneDB, where the latter is indicated with a “ + ” suffix. The mmu* data set represents a filtered version of the available mouse data in miRBase (mmu + represents mouse data from MirGeneDB) and the novel positive data sets we create present random values constrained to specific ranges (see “Methods” section).

The generated models were trained using human examples from miRBase, and they may not be applicable for other species in miRBase. In order to test how well these models generalize, they were used to predict all available pre-miRNAs from all 223 species available in miRBase (http://jlab.iyte.edu.tr/software/izmir) using the mirbase data set, data from MirGeneDB, and all negative data (Table 2). The Bentwich_NB_ model performed best with 97.53% correct predictions, followed by the Consensus_DT_ model with 96.71% (Supplementary Table 3). This analysis established the positive prediction rate of the trained models. Additionally, prediction on a subset of the positive data like hsa, mmu, and mmu* was tested, and Consensus_DT_ performed well in all cases (Table 3).

**Table 3:** Model performance summary

	Negative										Positive													
Model	NegHsa	Zou	Pseudo	NotBestFold	Shuffled	Chen	Pseudo_FR_	Pseudo_BQ_	Pseudo_AM_	Neg Rank	hsa	mmu	mmu*	mirbase	hsa_FR_	hsa_BQ_	hsa_AM_	mirgenedb	hsa+	mmu+	gga+	dre+	Pos rank	Total rank
Average_DT_	82	56	93	31	93	77	30	100	100	99	97	83	95	91	91	100	100	97	98	96	98	96	52	151
Consensus_NB_	89	52	86	24	96	77	53	100	100	97	86	82	93	89	100	100	100	96	96	93	98	97	84	181
Consensus_DT_	74	44	90	20	88	72	16	100	100	155	99	87	96	93	97	100	100	98	99	97	100	97	31	186
Ding_NB_	93	47	84	9	96	73	30	100	100	127	88	84	94	90	100	100	100	97	97	96	97	97	59	186
Average_NB_	92	58	89	95	97	82	86	100	100	50	83	77	91	87	99	100	100	94	95	91	96	95	148	198
Ng_DT_	74	64	89	13	91	77	31	100	100	118	89	80	93	88	85	100	100	96	96	94	98	97	100	218
Consensus Model	84	69	96	69	89	81	58	100	100	70	97	76	94	87	33	100	100	92	95	89	93	92	157	227
Batuwita_NB_	90	53	83	11	97	76	45	100	100	114	86	79	92	87	98	100	100	96	96	93	97	97	120	234
Bentwich_NB_	37	23	71	9	69	52	21	100	100	222	92	92	98	95	99	100	100	99	99	97	100	100	26	248
Ng_NB_	74	42	81	9	87	63	36	100	100	187	86	83	95	91	99	100	100	96	97	94	98	98	63	250
Mean	80	56	86	51	89	75	52	98	97		85	75	89	84	67	96	97	89	89	86	89	89		

Top ten models from the two machine learning algorithms generated for all 13 studies and 6 ensemble methods and their performance in respect to the 21 data sets examined in this study. The table is sorted by total rank which indicates the overall best performance considering all data sets equally. The minimum possible total rank is 21, and the maximum one is 672. Values are the prediction correctness (positive/negative) for the different data sets (Table 2). The complete results are provided in Supplementary Table 5 including highlighting similar to a heat map. The calculated mean refers to the complete results in Supplementary Table 5

It is important to assign positive examples correctly, but it is equally important to reject negative ones accurately. In order to establish how efficient non-miRNAs are rejected, nine data sets containing putative negative examples were acquired or established (Table 2). Xu_NB_ performed best for the combination of all negative data sets, followed by Xue_NB_ and Jiang_NB_. Interestingly, Bentwich_NB_ achieved very poor results for the prediction of pre-miRNAs (last rank) and the models performing well in negative data fail for positive data ranking in the bottom of the list (Supplementary Table 3). These results clarify that both positive and negative prediction rates need to be considered at the same time. Evaluating the tools according to the summed rank for both measures concurrently, the consensus models showed highest performance taking the first three ranks (Table 3). Overall, Average_DT_ performed best which is consistent with the receiver operating characteristic (ROC) analysis (Supplementary Fig. 4).

### Using izMiR on eukaryote genomes

Can computational models be applied to the analysis of large eukaryotic genomes is a question that needs careful evaluation. To test this, we analyzed the *Drosophila melanogaster* (dme) genome. We used izMiR models generated using human (hsa model) and Drosophila (dme model) hairpins. As a representative for human models, Average_DT_ was used to establish whether the known hairpins for dme can be found. Applying confidence thresholds of 0.96 and 0.84 to the 256 dme hairpins from miRBase, 183 hairpins were identified using the dme model while 144 hairpins were detected with the hsa model. As should be clear from previous works^22^ and from the filtered mouse results, it is unlikely that all dme hairpins in miRBase are true miRNAs.

From the genome-wide miRNA search in the dme chromosome 2L, we could not extract 16 out of the 56 drosophila hairpins mapped to the 2L chromosome in miRBase. Manual inspection revealed that the secondary structure predicted for the affected regions in the genome did not include structures suitable for pre-miRNAs. We discuss the validity of the 16 dme examples on the website for izMiR (http://jlab.iyte.edu.tr/files/izmir/HairpinAssessment.pdf). We note here that the dme model retrieved all of the hairpins we reject while the hsa model only detected 12 (missed ones: dme-mir-4943 (score: 0.33), dme-mir-288 (score: 0.24), dme-mir-1004 (score: 0.18), and dme-mir-4914 (score: 0.02). This analysis confirmed that both models can be used for the detection of pre-miRNAs in the dme chromosome 2L. For the remaining 40, we analyzed the prediction scores from Average_DT,_ and we set thresholds based on lower quartile values as 0.96 for dme model and 0.84 for the hsa model (Supplementary Fig. 9). With these cutoff scores, 25 of the miRNAs in the 2L chromosome were found in our extracted hairpins in both models’ predictions while dme-mir-275, dme-mir-9378, dme-mir-1006, dme-mir-966, dme-mir-967 hairpins, and dme-mir-125, dme-mir-275, dme-mir-9374, dme-mir-960, dme-mir-962, dme-mir-9c hairpins passed the hsa or dme models, respectively. Overall we predicted 17,455 candidate miRNAs with the dme model and 43,103 candidates with the hsa model out of 581,883 hairpins in the 2L chromosome (Supplementary Fig. 8). Among these sequences, there are likely to be redundant ones, as we confirmed using USEARCH for clustering of the ~290,000 sequences, which lead to the elimination of ~65,000 highly similar sequences from the pool. The threshold of the dme model is already very high, but the hsa model’s threshold can be further adjusted to reach a number of predictions suitable for experimental validation. For example, choosing a threshold of 0.99 for the hsa model leads to a mere 585 hairpins that need to be examined (Supplementary Table 6).

### Forming consensus is better than individual effort

**Fig. 2:**
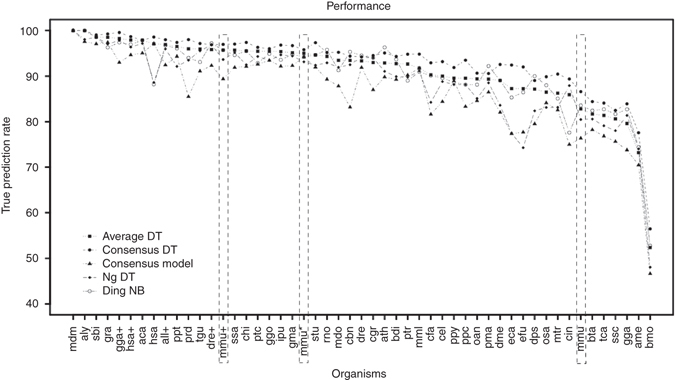
Generalization performance of izMiR. *Line plot* showing the true prediction rates (*y* axis) of hairpins from different organisms. Mmu* stands for filtered mouse hairpins from miRBase based on a minimum RPM value of 100 and mmu refers to all mouse hairpins without filtering while mmu + indicates mouse hairpins from MirGeneDB. Only organisms with a minimum of 200 hairpins in miRBase were selected for this plot. Results for all organisms in miRBase are available for download from our web page: http://jlab.iyte.edu.tr/izmir. The *lines* do not indicate a mathematical relationship and are only added for simplifying visual tracking

Deciding which of the 13 methods to use can be a daunting task. Initially, tools need to be acquired, but some may not be available or functional (Table 1). Then these tools need to be compared and their settings need to be optimized for the given problem, which is a complicated and time-consuming process. With the izMiR framework, we solved all of these problems by providing a working implementation of all 13 tools. Furthermore, for all these tools we analyzed their performance on all known pre-miRNAs in miRBase, all previously used negative data sets, and novel ones. For different models generated from different classifiers (DT, NB, and SVM) Ding, NG, and Chen perform best (Supplementary Table 1), but only Xue and Lopes can be readily discarded as alternatives while among the others none significantly outperforms all others (Fig. 1). To overcome this decision-making processes, we developed ensemble methods, and notably, Average_DT_ outperforms all other tools (Table 3). Applying this methodology to all available data on miRBase shows that it performs extremely well for most species, including plants (Fig. 2).

We were not able to determine a relationship between any parameters like evolutionary distance and the true prediction rate (TPR). However, spikes in the graph may indicate the presence of pre-miRNAs which are erroneously named such. This conclusion is further supported by the difference in mmu and mmu* performance (Fig. 2) as well as by the fact that dme is in the lower accuracy range, and we showed that at least 1% of the pre-miRNAs are unlikely to be correct and finally by the spike in human for which we previously showed that some of the pre-miRNAs are unlikely to be true^22^.

## Discussion

MiRNAs are of major interest as they can be disease markers, therapeutics, or agents to increase agricultural productivity. Since it is convoluted to detect novel miRNAs experimentally, implementation and use of computational tools for miRNA analysis has gained importance. All available tools for pre-miRNA detection discussed here (Table 1) employ machine learning for which training data quality is of crucial importance.

Unfortunately, it is currently impossible to establish a true negative data set and, therefore, the quality of available negative data sets is hard to assess^23, 24^. The Zou, NotBestFold, and pseudo_FR_ data sets are difficult to solve for most algorithms (Table 3). This may stem from the use of coding sequences for the construction of Zou, which may in part contain pre-miRNAs^24^. Our NotBestFold data set consist of pre-miRNAs from miRBase, but suboptimal folds were used in its construction, so that approaches focusing on sequence will still be able to name them pre-miRNAs, whereas those using structure-related features may not. Finally, pseudo_FR_ was generated using random number generation within the maximum range in the pseudo data set, which apparently shifts the distribution such that the data set becomes much harder to solve this is supported by the better performance of pseudo_BQ_ and pseudo_AM_ which were generated using smaller intervals (see “Methods” section). The minimum free energy and *p* value distributions among data sets show that the ones for Zou and Chen are quite similar to the positive examples (hsa, mmu), which may explain low performance of models and may need further scrutiny (Supplementary Figs. 6, 7).

Positive training data is generally taken from miRBase, and while it is clear that having true negative data is not currently possible, our previous analysis^23^ and our results for mouse (Table 3, Fig. 2) indicate that positive data may also need further scrutiny. A simple filtering approach based on removing mouse hairpins with low transcriptomic evidence (read per million counts of less than 100) led to a 10% difference for correct prediction rate (Fig. 2). Using only mouse hairpins from MirGeneDB further improved the results but led to a wider spread among model performance. Considering that the proteins taking part in miRNA biogenesis are conserved in most eukaryotes^23, 25^, increasing the positive data quality may help produce better models that can be applied to a wide range of organisms as izMiR exemplifies (Fig. 2). To enable future studies in this regard, we combined the building blocks for a machine learning approach into a unified, comprehensive, yet adjustable and extensible data analytics workflow that is publicly available at http://jlab.iyte.edu.tr/software/izmir.

All relevant studies in the field of ab initio pre-miRNA detection were compared impartially, and it was established that Bentwich_NB_ most correctly classified positive data achieving 97.53% TPR, closely followed by Consensus_DT_ with a TPR of 96.71% (Supplementary Table 5). For negative data, Xu_NB_ performed best, achieving a true negative rate (TNR) of 94.66% followed by Xue_NB_ and Jiang_NB,_ which achieved a TNR of 94.66% and 93.68%, respectively. More important than these individual achievements is whether a model performs well for TPR and TNR at the same time. The best average correct prediction rate was achieved by Average_NB_ (90.72%) followed by Gao_NB_ (86.20%), but these numbers may be affected by outliers so that we base our decisions on the lowest sum of ranks for all data sets. Among the 13 studies evaluated, Burgt_Dt_ and Xue_DT_ had the lowest sum of ranks (tie), but most of our ensemble methods ranked in the top ten. When not considering ensemble methods, we advise to use either Jiang_NB_ or Ding_NB_, which on average perform similarly well. Taking into account ensemble methods, Average_DT_ had a lower sum of ranks than Consensus_NB_ and considering the trained models (Supplementary Figs. 4, 5), Average_DT_ performed best and reached an area under the ROC curve of 0.99 while Average_NB_ only achieved 0.93 and only ranked fifth (Table 3). Therefore, we suggest using Average_DT_ for detection of pre-miRNAs as a default. Homology-based pre-miRNA detection is not discussed in this work since the automatic selection of homologs from a suitable evolutionary distance in a framework like izMiR is currently not possible. However, homology-based features can seamlessly be integrated into izMiR allowing specialists to extend the framework accordingly. We suggest, however, to separate these tasks and first perform pre-miRNA detection using izMiR followed by an evolutionary assessment of the detected hairpins by using tools like RNAmicro^26^.

The izMiR framework contains all 13 individual models and their 6 ensemble methods. For the consensus models, we assigned equal weights to all studies although they did not perform equally well, which may be improved upon in the future. Moreover, our model selection is based on the highest accuracy scores, which may not be the most reliable method in all cases. To overcome this issue, we provided other scores like the F-measure and Youden’s index to aid alternative model selection strategies. We present the state of the art in ab initio miRNA detection, introduce methods to combine available models synergistically, and provide an implementation for all analyzed studies as well as for our consensus methods. The developed framework further simplifies the generation of new classifiers, and enables their comparison to the state of the art, thus accelerating future developments.

How well do models trained for one species generalize to all other species available on miRBase? Unfortunately, this question cannot be directly answered when simply applying the models to all available data from all species (Fig. 2; http://jlab.iyte.edu.tr/software/izmir). We conclude that the quality of the data available in miRBase determines the TPR and that any other factors are likely of less importance (Fig. 2). Thus, Average_DT_ should be used for the detection of pre-miRNAs from any species. The izMiR framework allows the generation of new models which can be more effective for particular scenarios, for example, a selected species. It should be noted that although the number of different types of pre-miRNAs is unknown, we experienced difficulties when training models with less than 1000 positive examples. These 1000 examples further need to be very good, for which high RPMs seems to be one indication (e.g.: RPM > 100), but the best would be to manually review all examples and remove the ones that seem unlikely. Therefore, we suggest to only train new models for very specific scenarios. The izMiR framework can also be used to develop new approaches and ensemble methods, and compare them to the state of-the art. We believe that there is still room for improvement and encourage the use of izMiR for the development of new approaches.

Millions of candidate hairpins exist in eukaryotic genomes, but the unequaled performance of the Average_DT_ model facilitates the computational detection of pre-miRNAs in even larger eukaryotic genomes, which is of great interest since they are hard to detect experimentally. The application of our Average_DT_ model using the dme genome showed that although our models are generated on human pre-miRNAs, they can perform well in other organisms (Table 3, Fig. 2). Through manual inspection of the *Drosophila* hairpins from miRBase, which were not classified as pre-miRNAs by our consensus models, such as dme-mir-4914, dme-mir-4912, and dme-mir-9382, we found that they have questionable secondary structures which do not conform to expectations for pre-miRNAs recognizable for proteins in the miRNA biogenesis pathway (detailed assessments for all missed pre-miRNAs are available on the izMiR website). Evaluating the 56 dme pre-miRNAs of the chromosome 2L available in miRBase reveals that the human-trained izMiR model generally gave lower scores to the dubious hairpins than the *Drosophila*-trained izMiR model. Applying both models to the candidate hairpins, 32 passed the hsa model at a confidence of 0.84 while 43 passed the dme model at a 0.96 confidence threshold. All but two of the dubious dme models are excluded using the hsa model whereas the dme model includes six of them. Further adjustment of the hsa model threshold can reduce the number such that experimental confirmation of all predictions becomes possible. However, we suggest to include instead targeting information^27^ and expression information^15, 28^, which further reduces the number of candidates and allows experimental validation of thusly filtered candidates. Pre-miRNAs are not the final mature form of miRNAs. izMiR does not predict mature miRNAs, but after detecting high-quality pre-miRNAs using izMiR, mature miRNAs can be situated within them using existing tools like MatureBayes^29^ and MaturePred^30^.

izMiR is instrumental for pre-miRNA detection from next generation sequencing data. With the advent of next-generation sequencing (NGS), small RNAs like miRNAs have been successfully detected using read mapping to a reference genome or to a de novo assembled transcriptome. Computational pipelines for the detection of pre-miRNAs from NGS data have been developed, and all contain a module which checks whether the mapped mature sequences are part of a viable pre-miRNA^31–33^. These pre-miRNA tests (some of which are quite basic) employ features describing pre-miRNAs and/or read mapping, but have not been rigorously tested. Separating such tests into pre-miRNA detection using izMiR and filtering by read-mapping statistics would ensure that at least the pre-miRNA detection functionality has been thoroughly assessed.

In conclusion, izMiR allowed the impartial comparison of existing ab initio pre-miRNA detection tools, enabled the development of new and the integration of existing tools, was easily trained with novel data, was applicable to a wide range of species using Average_DT_, and facilitated the detection of pre-miRNAs in large eukaryotic genomes.

## Methods

### Data sets for machine learning

Positive examples for machine learning were retrieved from miRBase^34^, the de facto standard for positive training data used in ab initio pre-miRNA prediction (release 21). We performed filtering operations like removing hairpins with identical sequences which reduced the overall amount of positive examples to 1828 human pre-miRNAs for the human training data set. For prediction, however, unfiltered miRBase data were used. In the *Drosophila melanogaster* analysis, we also generated models by using 256 hairpins from miRBase as the positive dataset. Moreover, all of the miRNAs listed in miRGeneDB (v1.1)^35^ were included for prediction as species specific and as one combined data set.

Similar to an idea we outlined in a previous paper in the field of proteomics^36^, it is vital that training and testing data sets become more challenging with increased accuracy of trained models. Therefore, we used a variety of negative data sets in order to enable comparison among detection methods and to establish the current state of the art:

- *Pseudo*: previously published by Ng^37^, used for learning and prediction since the data set is challenging but not unsolvable^38^ and, therefore, a good basis for creation of robust models (negative data; 8492 hairpins)
- *Shuffled*: derived from shuffling sequences of human positive data from miRBase, used for prediction (negative data; 1423 hairpins)
- *NotBestFold*: created by not using the best fold proposed by RNAFold^39^ for human hairpins from miRBase, used for prediction (negative data; 1881 hairpins)
- *NegHsa*: previously published by Gudys^40^, used for prediction (negative data; 68,048 hairpins^*^)
- *Zou*: previously published by Zou et al.^24^, used for prediction (negative data; 14,246 hairpins)
- *Chen*: previously published by Chen et al.^41^, composed of samples from Pseudo and Zou, used for prediction (negative data; 3054 hairpins)
- *mirgenedb*: all miRNAs available in miRGeneDB (v1.1)^35^, used for prediction (positive data; 1434 hairpins)
- *hsa + : Homo sapiens* miRNAs available in miRGeneDB (v1.1)^35^, used for prediction (positive data; 523 hairpins)
- *mmu + : Mus musculus* miRNAs available in miRGeneDB (v1.1)^35^, used for prediction (positive data; 395 hairpins)
- *gga + : Gallus gallus* miRNAs available in miRGeneDB (v1.1)^35^, used for prediction (positive data; 229 hairpins)
- *dre + : Danio rerio* miRNAs available in miRGeneDB (v1.1)^35^, used for prediction (positive data; 287 hairpins)
- *hsa*_*FR*_: created by generating random numbers between minimum and maximum values of each feature in human miRNA data set based on miRBase, used for prediction (positive data; 5000 hairpins)
- *hsa*_*BQ*_: created by generating random numbers between lower quartile and upper quartile values of each feature in human miRNA data set based on miRBase, used for prediction (positive data; 5000 hairpins)
- *hsa*_*AM*_: created by generating random numbers between 40 quantile and 60 quantile values of each feature in human miRNA data set based on miRBase, used for prediction (positive data; 5000 hairpins)
- *pseudo*_*FR*_: created by generating random numbers between minimum and maximum values of each feature in pseudo data set, used for prediction (negative data, 5000 hairpins)
- *pseudo*_*BQ*_: created by generating random numbers between lower quartile and upper quartile values of each feature in pseudo data set, used for prediction (negative data; 5000 hairpins)
- *pseudo*_*AM*_: created by generating random numbers between 40 quantile and 60 quantile values of each feature in pseudo data set, used for prediction (negative data; 5000 hairpins)

^*^Note, that the original data set of NegHsa (http://adaa.polsl.pl/agudys/huntmi/huntmi.htm) contains many duplicate identifiers and we forced them to be unique, thereby reducing the amount of data from 87,000 to ~68,000 examples.

### Hairpin extraction from genome data

In order to extract hairpins from a genome, it was first divided into 500 nt fragments with 250 nt overlaps, and then, the sequence was converted to RNA (T→U) as well as reverse complemented for the template strand. All secondary structures were predicted using RNAfold^39^, and regular expressions were used to extract all structures that resembled a hairpin (stem with at least three consecutive matches and a terminal loop with at least three nucleotides). The resulting hairpins were filtered according to human hairpin length distribution on miRBase, and duplicate sequences were removed. All features for pre-miRNA detection were calculated for the remaining hairpins and analyzed with the trained human models according to the protocol we deposited on Nature Protocol Exchange (http://www.nature.com/protocolexchange/protocols/4919).

The human genome (GRCh38, DNA, primary assembly) contains 12,399,093 fragments from which 108,788,895 putative hairpins for one strand and 108,276,240 hairpins for the other were extracted and filtered based on hairpin length (between 36 and 180; representing the smallest and the longest human stem loops in miRBase). After removing duplicate sequences from the 34,856,229 length-filtered hairpins, 27,932,492 putative pre-miRNA sequences remained. The same filtering approach resulted in 28,074,667 hairpins for the other strand.

The dme genome (BDGP6 genome assembly) was fragmented into overlapping (250 nt) fragments of 500 nt in length (575,896 fragments). RNAFold was used to create the secondary structure of all fragments, and regular expressions were used to extract all structures remotely similar to hairpins leading to about 5 million hairpins per strand. Hairpins with less than 30 nucleotides were filtered leaving ~2 million hairpins per strand. The chromosome 2L contained about 360,000 hairpins per strand, and after removing duplicates, all hairpin features were calculated for this subset of putative pre-miRNAs (about 290,000 per strand).

For human, we would have to calculate about 700 features for all putative hairpins which could take several months on a high-end personal computer. Processing such a large data set was beyond the scope of this study, and we selected the 2L chromosome of dme to exemplify the effectiveness of our pre-miRNA detection method even for evolutionary distant species by employing a human-trained izMiR and a Drosophila-trained izMiR model.

### Features for pre-miRNA parameterization

For machine learning, pre-miRNAs need to be parameterized, and many features have been described in the literature (Table 1). The features that have been used for pre-miRNA prediction can be divided into four categories; sequence-based, structural, probability-based, and thermodynamic; although some features can be categorized into more than one of these basic categories. For this study, we implemented ~900 features, covering the features used or proposed in the 13 studies evaluated here (Table 1), published in other studies or designed by us. Some of the proposed features were ambiguously defined which opened room for their interpretation, and we implemented them to the best of our understanding. There are various methods to calculate these features^42^, and we define the features used in pre-miRNA analyses in another work^25^ and on our web page: http://jlab.iyte.edu.tr/software/izmir.

### Training models for pre-miRNA detection

**Fig. 3:**
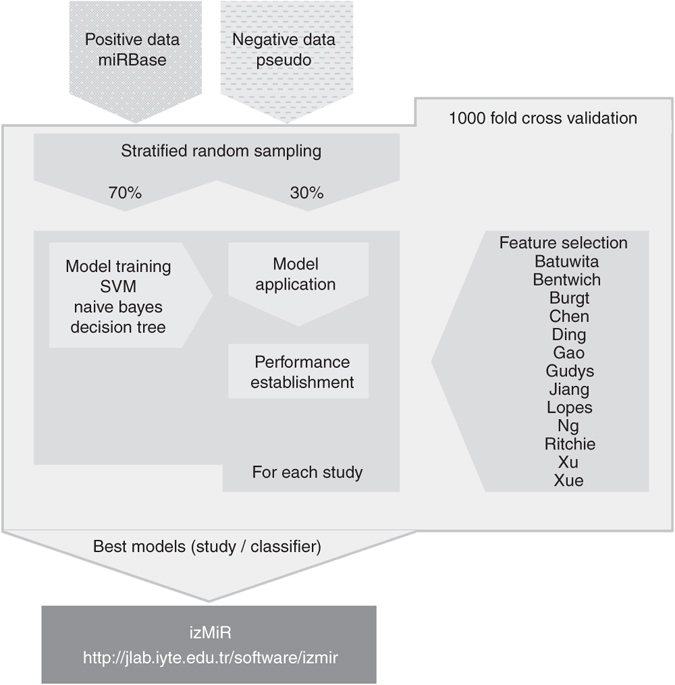
Model training workflow. Filtered human miRNA hairpins from miRBase served as positive data and pseudo hairpins for negative data. Each data set is randomly sampled individually; 70% of positive data and the same number of negative examples were used during 1000-fold Monte Carlo cross validation (MCCV) ^30^. The remaining 30% of positive data and the same number of negative examples are used for testing the model. In the end, the best models for naïve Bayes and decision tree were stored for prediction in PMML format while SVM performance was not stored as a PMML model due to limitations of the available SVM implementation

In machine learning, first, a model is trained based on examples (here: positive and negative examples). There are many training and testing schemes used and different studies performing ab initio pre-miRNA detection used different approaches. It is our apprehension that high ratios of training ( >= 90%) to testing data are only useful when the amount of data is severely limiting. We believe that such schemes would overestimate the actual model performance. Since the availability of data is indeed limited, we settled for a training-testing scheme of 70–30% (Fig. 3). Classification for model generation and predictions on data sets were performed using KNIME^43^ which is a workflow management and data analytics platform.

For classification (Fig. 3), since there was a big class imbalance among the data which may influence the overall performance significantly^25^, it was essential to design an efficient strategy for learning. The popular approaches like *k*-fold cross-validation and leave-one-out have many shortcomings^44, 45^. Due to this, we decided to sample positive and negative data separately. After random sampling equal amounts of examples from the positive and negative data pools, examples were randomly divided into training (70%) and testing groups (30%). The input data were used to train three classifiers NB, DT, and support vector machine SVM, and their performance scores and PMML models (best only) were stored for each iteration. Through 1000 iterations of the sampling and learning procedure, we obtained the best PMML models for NB and DT. To evaluate model performance, the following measures were recorded: recall, precision, sensitivity, specificity, F-measure, accuracy, Cohen’s kappa, and Youden’s index. For SVM, Weka LibSVM (3.7) was used since it was fast comparing to other SVM implementations available in KNIME. However, since Weka models’ PMML outputs were not compatible with our system, we could not save SVM models, but, produced scores during learning and testing to enable comparisons with NB and DT classifiers. The training workflow guarantees that each study and classifier receives identical data in each iteration, ensuring a fair comparison. The training workflow is publicly available on our web page: http://jlab.iyte.edu.tr/software/izmir and further explained in detail on Nature Protocol Exchange (http://www.nature.com/protocolexchange/protocols/4919).

### Predicting pre-miRNAs with izMiR

Many studies present their findings of model training but fail to actually provide the model so that it can be used for prediction. In this study, we provide NB and DT models for each study as well as some consensus models combining all studies.

For prediction, the best DT and NB models for each study were loaded into another workflow which was designed to apply these models to input data and to associate scores to predictions in order to allow detection of pre-miRNAs. The individual PMML models for each classifier, which were produced during training can be used for prediction of pre-miRNAs in our system developed in KNIME (http://jlab.iyte.edu.tr/software/izmir). Additionally, some consensus schemes were devised which combine the power of individual studies to improve classification performance. These consensus approaches are also available in the izMiR framework we provide.

In order to obtain a consensus result, equal weights were given to each model, and a given sequence was labeled as “miRNA” by Consensus_DT_ and/or Consensus_NB_ models if it was predicted as miRNA in seven or more studies (majority vote).

For ConsensusRule based prediction, the average of DT and NB prediction scores for each putative pre-miRNA were taken into consideration; if average DT score or average NB score was larger than 0.89, then it was labeled as “miRNA”. Conversely, if average DT score or average NB score was less than 0.5, it was labeled as “negative”. Finally, the remainder was labeled as “candidate” pre-miRNA.

Average_DT_ and Average_NB_-based predictions were performed in a similar manner to ConsensusRule. The average of DT and NB prediction scores for each putative pre-miRNA were taken into consideration; if their average value was smaller than 0.5, then it was labeled as “negative”, otherwise it was labeled as “miRNA”.

For ConsensusModel prediction, scores were obtained for human miRNAs and pseudo data set by using the models from each study. Then, these scores, ranging between 0 and 1, were used to train a multi-layer perceptron classifier (following the same procedure as described for learning). The model with the highest accuracy and F-measure was stored for later use.

The input data for prediction was applied to all individual and consensus models described above, and the numbers of entries predicted as “miRNA”, “negative”, and “candidate” were returned for all of them. TPR and TNR) were calculated as performance measures according to the following expressions:

TPR = (number of hairpins correctly classified as “pre-miRNA”/number of overall hairpins) × 100

TNR = (number of hairpins correctly classified as “negative”/number of overall hairpins) × 100

### ROC curves to estimate model performance

The models with the highest accuracy score for DT and NB were applied to human pre-miRNAs from miRBase and pseudo negative data set to estimate true and false positive rates and to construct ROC curves. Along with the study-based models, Average_DT_ and Average_NB_, referring to two of our consensus methods, were analyzed in this manner (Supplementary Figs. 4, 5).

### Data availability

The data sets generated during and/or analyzed during the current study are available in the izMiR repository, http://jlab.iyte.edu.tr/software/izmir.

## Electronic supplementary material

Supplementary Information
