# Supplementary material for: On the performance of pre-microRNA detection algorithms
Source: Nat Commun. 2017 Aug 24;8:330. doi: 10.1038/s41467-017-00403-z (PMC5571158; doi:10.1038/s41467-017-00403-z)
Supplement: Supplementary file 1 — Supplementary Information [file 41467_2017_403_MOESM1_ESM.pdf]

Description of Supplementary Files

File Name: Supplementary Information
Description: Supplementary Figures and Supplementary Tables

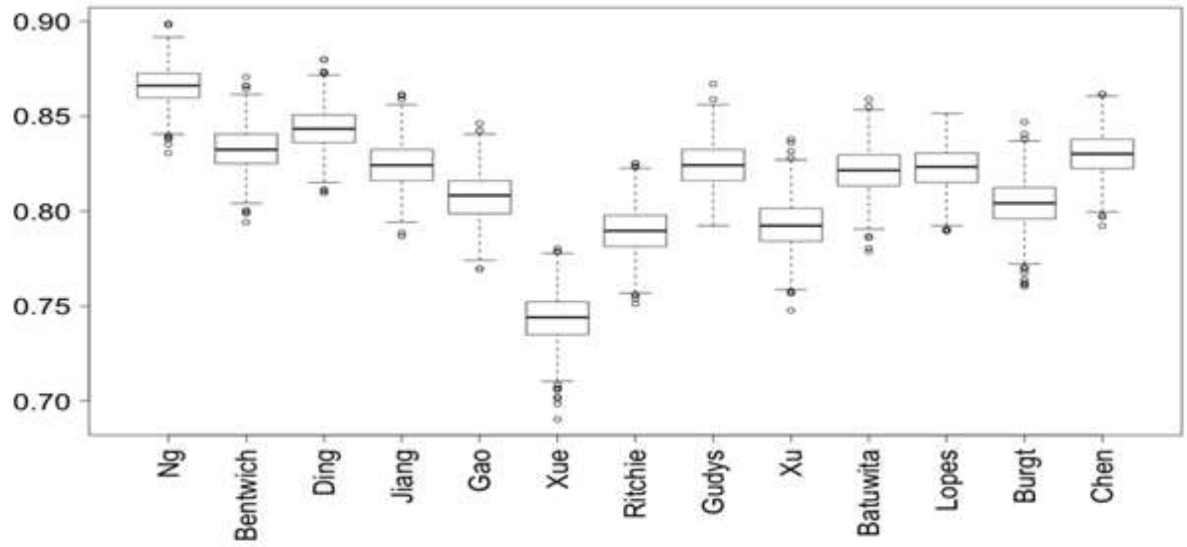

Supplementary Figure 1. Classifier accuracy distribution. Box-whisker plots showing the accuracy distribution for decision tree models from 13 studies using 1000 fold MCCV. Ng performs significantly better than all other studies.

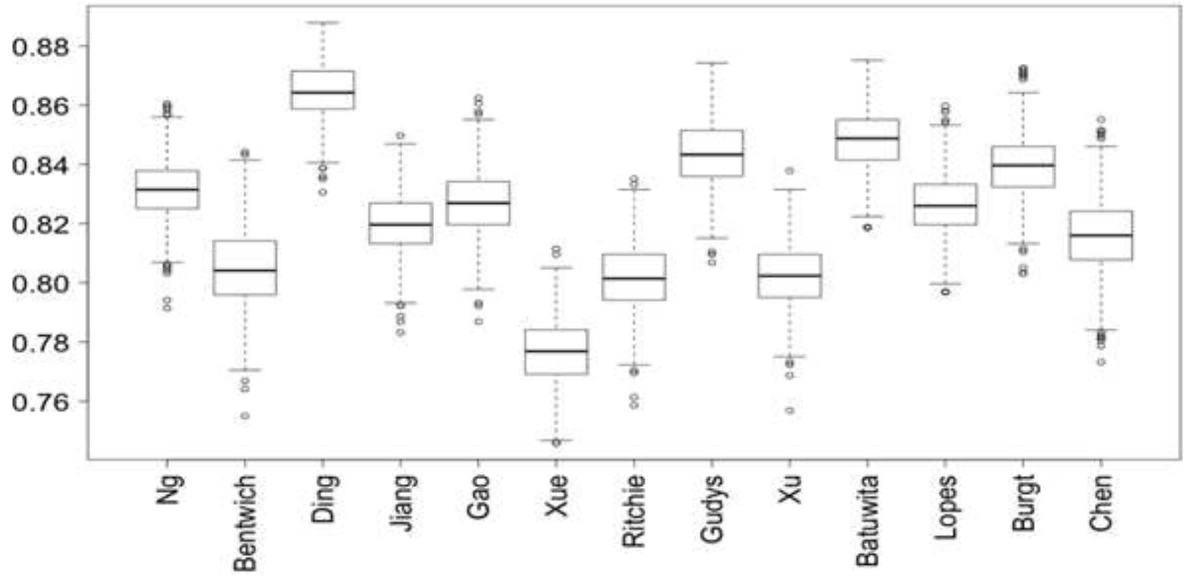

Supplementary Figure 2. Classifier accuracy distribution. Box-whisker plots showing the accuracy distribution for naïve Bayes models from 13 studies using 1000 fold MCCV. Ding performs significantly better than all other studies.

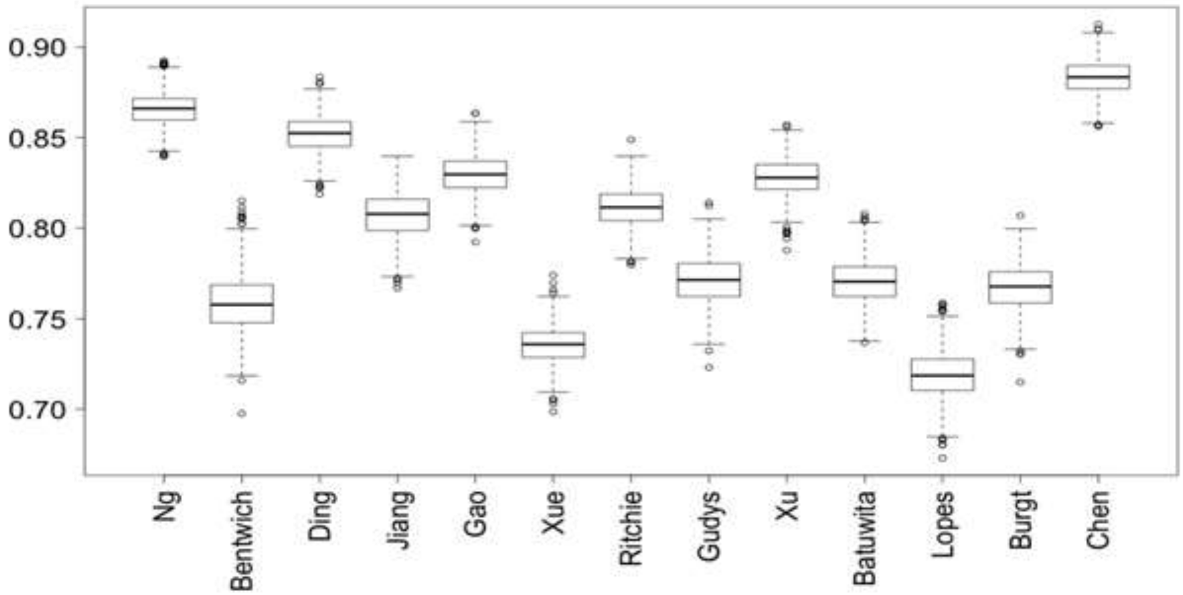

Supplementary Figure 3. Classifier accuracy distribution. Box-whisker plots showing the accuracy distribution for support vector machine models from 13 studies using 1000 fold MCCV. Chen is clearly better than all other studies, which is not the case for decision tree (Supplementary Figure 1) and naïve Bayes (Supplementary Figure 2) while Ng and Ding perform well for all classifiers (cf. Supplementary Table 1).

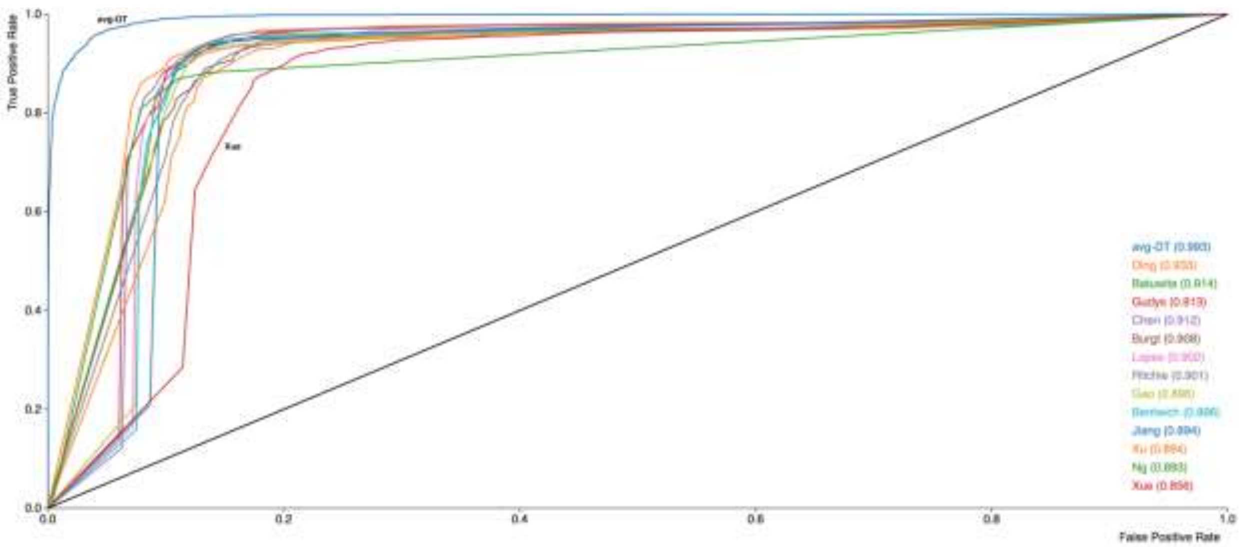

Supplementary Figure 4. ROC curve graph. ROC curve for the best Decision Tree models applied to human miRNAs and pseudo datasets. The Average$_{DT}$ model performs significantly (more than two standard deviations) better than all individual studies.

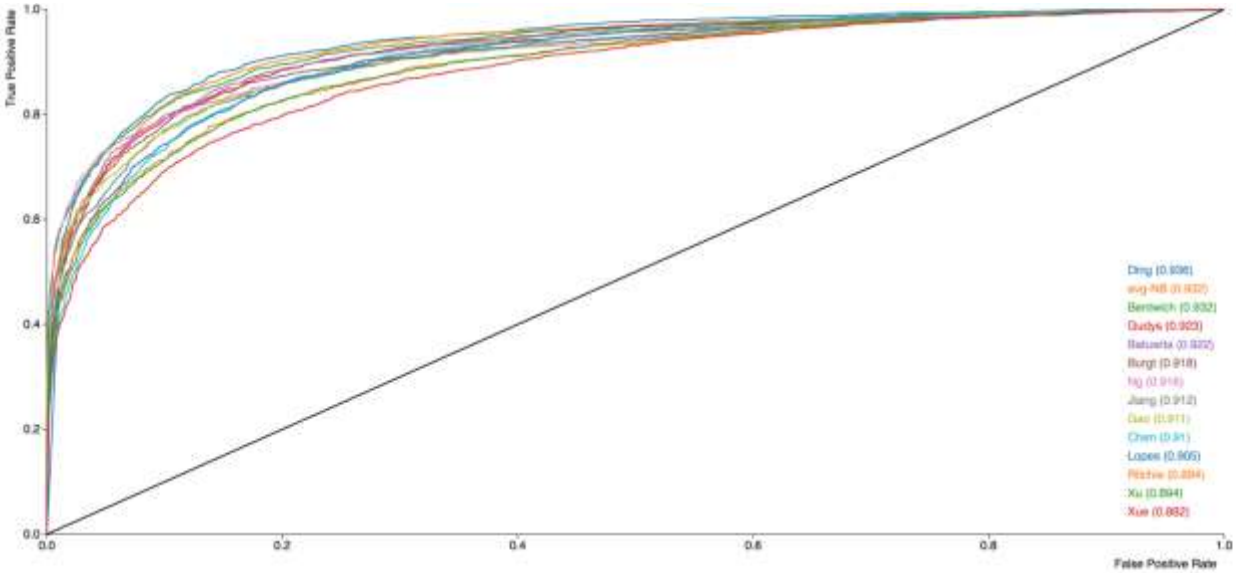

Supplementary Figure 5. ROC curve graph. ROC curve for the best naïve Bayes models applied to human miRNAs and pseudo datasets. Similar to previous observations (Supplementary Figure 2, Supplementary Table 2) NB results are very similar and have a very low spread. Average$_{NB}$, while not the best model has only 0.04 less AUC. Best AUC is comparable to second best AUC for DT (Supplementary Figure 4).

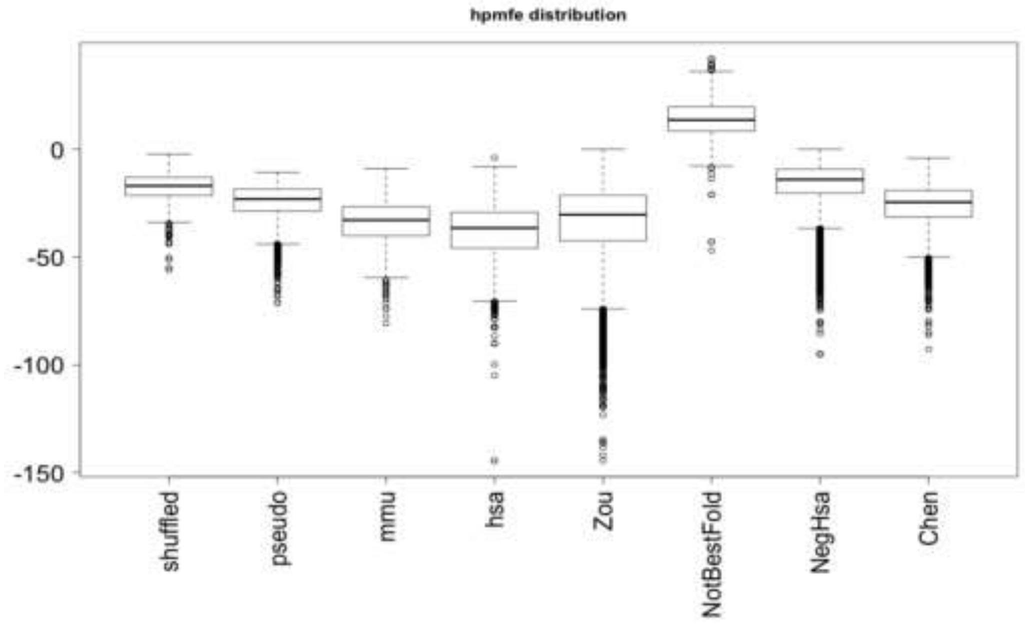

Supplementary Figure 6. Minimum free energy distribution. Box-plot graph showing the distribution of hairpin minimum free energy values (hpmfe, calculated by using RNAFold) in various data sets. Mmu and hsa include only positive examples while all other datasets contain negative ones.

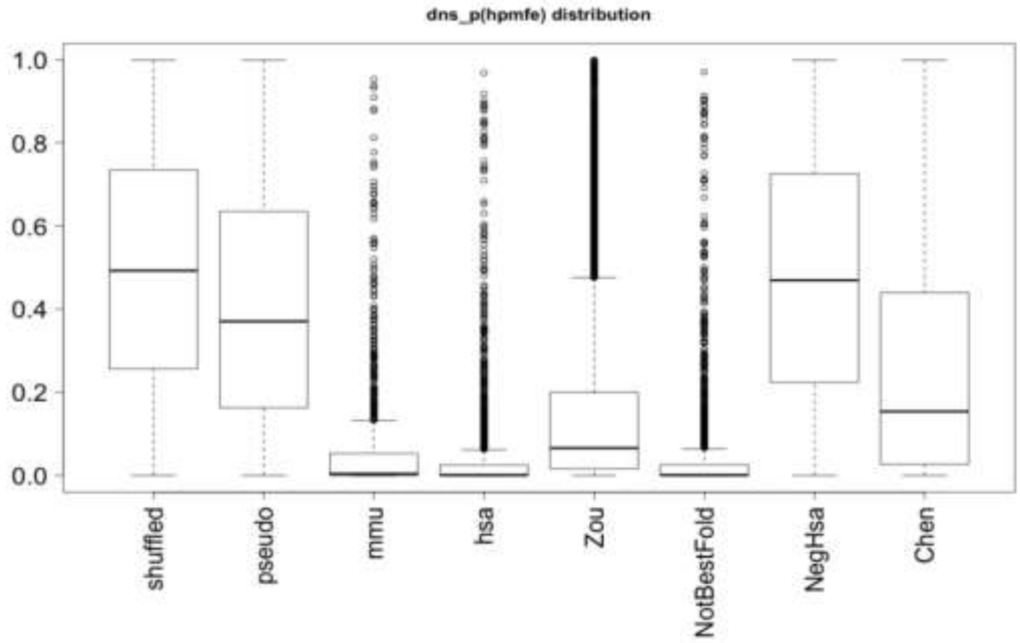

Supplementary Figure 7. P value distribution. Box-plot graph showing the p value distribution of hairpin minimum free energy values obtained through dinucleotide shuffling, in various data sets. Mmu and hsa include only positive examples while all other datasets contain negative ones.

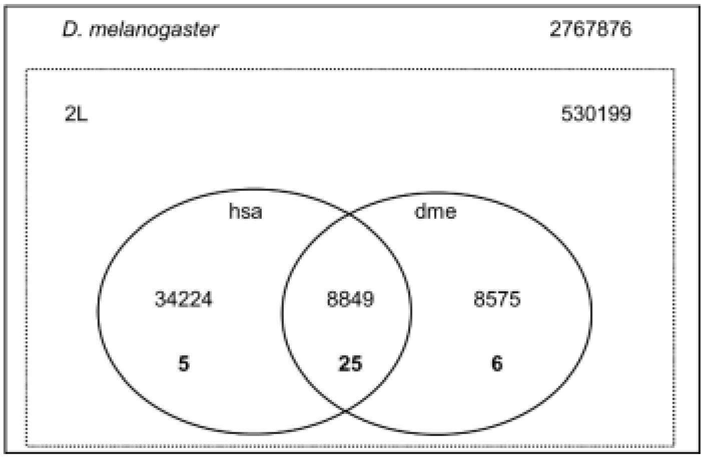

Supplementary Figure 8. Number of predicted hairpins from *Drosophila melanogaster*. Circles include number of hairpins passing confidence thresholds in hsa and dme models. Numbers in bold characters indicate miRBase hairpins extracted and passed the thresholds.

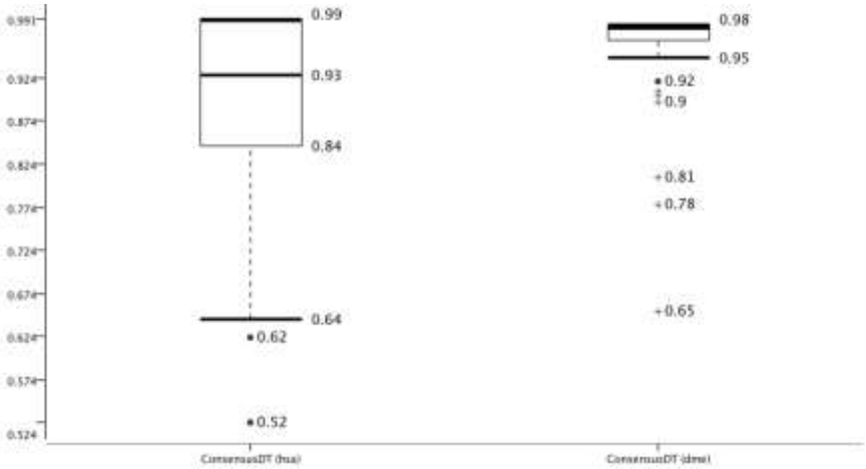

Supplementary Figure 9. Prediction score distribution. Box plot graph showing the prediction score distribution for the consensus $Average_{DT}$ classifier for 40 hairpins extracted from the 2L chromosome from *Drosophila melanogaster*. Lower quartile values 0.84 and 0.96 are used as thresholds for prediction analysis.

Study	DT	NB	SVM	Sum	Range
**Ding**	2	1	3	6	2
**Ng**	1	5	2	8	4
**Chen**	4	9	1	14	8
**Gudys**	6	4	8	18	4
**Batuwita**	8	2	9	19	7
**Jiang**	5	8	7	20	3
**Gao**	9	7	5	21	4
**Burgt**	10	3	10	23	7
**Xu**	11	10	4	25	7
**Bentwich**	3	12	11	26	9
**Lopes**	7	6	13	26	7
**Ritchie**	12	11	6	29	6
**Xue**	13	13	12	38	1

Supplementary Table 1: Study rank in respect to accuracy density. The ranks were manually assigned based on the Supplementary Figures 1-3. Ng, Ding, and Chen perform best for one algorithm, DT, NB, and SVM, respectively. Overall, Ding is most independent of the algorithm used and fares best in this comparison whereas Bentwich is most strongly affect by the classifier, closely followed by Chen.

	Accuracy		
	DT	NB	SVM
**Min**	0.69	0.75	0.67
**Average**	0.82	0.82	0.80
**Max**	0.90	0.89	0.91
**Spread**	0.21	0.14	0.24

Supplementary Table 2: Classifier comparison. Manually extracted overall accuracy distribution for Supplementary Figure 1-3. While SVM has the lowest accuracy, it also has the highest accuracy. NB has the lowest spread, but DT has a higher accuracy than NB while having a lower spread than SVM.

Negative Data	Positive Data	All Data
Xu_{NB}	$Bentwich_{NB}$	$Average_{NB}$
Xue_{NB}	$Consensus_{DT}$	Gao_{NB}
$Jiang_{NB}$	$Average_{DT}$	$Consensus_{NB}$
$Ritchie_{NB}$	$Ding_{NB}$	$Average_{DT}$
ConsensusRule	Ng_{NB}	ConsensusModel
Gao_{NB}	$Consensus_{NB}$	$Jiang_{NB}$
$Average_{NB}$	$Gudys_{NB}$	Xu_{DT}
$Burgt_{NB}$	$Batuwita_{NB}$	$Batuwita_{NB}$
ConsensusModel	Ng_{DT}	$Ding_{NB}$
Xu_{DT}	$Lopes_{NB}$	$Ritchie_{NB}$
Gao_{DT}	$Lopes_{DT}$	$Consensus_{DT}$
Xue_{DT}	$Bentwich_{DT}$	Gao_{DT}
$Ritchie_{DT}$	$Average_{NB}$	Ng_{DT}
$Chen_{NB}$	$Gudys_{DT}$	$Chen_{NB}$
$Consensus_{NB}$	$Batuwita_{DT}$	$Gudys_{NB}$
$Burgt_{DT}$	$Jiang_{DT}$	$Lopes_{NB}$
$Average_{DT}$	$Chen_{DT}$	ConsensusRule
$Batuwita_{NB}$	$Chen_{NB}$	Xu_{NB}
$Jiang_{DT}$	Xu_{DT}	$Jiang_{DT}$
$Lopes_{NB}$	$Ding_{DT}$	Ng_{NB}
Ng_{DT}	ConsensusModel	Xue_{NB}
$Ding_{NB}$	Gao_{DT}	$Batuwita_{DT}$
$Gudys_{NB}$	Xue_{DT}	$Gudys_{DT}$
$Batuwita_{DT}$	$Burgt_{DT}$	Xue_{DT}
$Chen_{DT}$	$Ritchie_{DT}$	$Burgt_{NB}$
$Ding_{DT}$	Gao_{NB}	$Bentwich_{DT}$
$Gudys_{DT}$	$Burgt_{NB}$	$Chen_{DT}$
$Consensus_{DT}$	$Jiang_{NB}$	$Ritchie_{DT}$
Ng_{NB}	$Ritchie_{NB}$	$Burgt_{DT}$
$Bentwich_{DT}$	ConsensusRule	$Lopes_{DT}$
$Lopes_{DT}$	Xu_{NB}	$Ding_{DT}$
$Bentwich_{NB}$	Xue_{NB}	$Bentwich_{NB}$

Supplementary Table 3: Study rank in respect to datasets. The average for prediction of all positive datasets and the average for the various negative datasets used in this study were calculated. The Models were ranked according to their performance on these datasets and the average for positive and negative data. The last column shows models ranked by all data.

	Negative										Positive													
**Model**	**NegHsa**	**Zou**	**Pseudo**	**NotBestFold**	**Shuffled**	**Chen**	**$Pseudo_{FR}$**	**$Pseudo_{IBQ}$**	**$Pseudo_{AM}$**	**Neg Rank**	**hsa**	**mmu**	**mmu***	**mirbase**	**hsa_{FR}**	**hsa_{BQ}**	**hsa_{AM}**	**mirgenedb**	**hsa+**	**mmu+**	**gga+**	**dre+**	**Pos Rank**	**Total Rank**
**$Average_{DT}$**	82	56	93	31	93	77	30	100	100	99	97	83	95	91	91	100	100	97	98	96	98	96	52	151
**$Consensus_{NB}$**	89	52	86	24	96	77	53	100	100	97	86	82	93	89	100	100	100	96	96	93	98	97	84	181
**$Consensus_{DT}$**	74	44	90	20	88	72	16	100	100	155	99	87	96	93	97	100	100	98	99	97	100	97	31	186
**$Ding_{NB}$**	93	47	84	9	96	73	30	100	100	127	88	84	94	90	100	100	100	97	97	96	97	97	59	186
**$Average_{NB}$**	92	58	89	95	97	82	86	100	100	50	83	77	91	87	99	100	100	94	95	91	96	95	148	198
**Ng_{DT}**	74	64	89	13	91	77	31	100	100	118	89	80	93	88	85	100	100	96	96	94	98	97	100	218
**Consensus-Model**	84	69	96	69	89	81	58	100	100	70	97	76	94	87	33	100	100	92	95	89	93	92	157	227
**$Batuwita_{NB}$**	90	53	83	11	97	76	45	100	100	114	86	79	92	87	98	100	100	96	96	93	97	97	120	234
**$Bentwich_{NB}$**	37	23	71	9	69	52	21	100	100	222	92	92	98	95	99	100	100	99	99	97	100	100	26	248
**Ng_{NB}**	74	42	81	9	87	63	36	100	100	187	86	83	95	91	99	100	100	96	97	94	98	98	63	250
**$Gudys_{NB}$**	87	48	81	14	96	76	27	100	100	140	87	80	92	88	100	100	100	95	95	93	97	96	113	253
**$Lopes_{NB}$**	89	54	80	9	96	73	39	100	100	134	86	79	93	86	98	100	100	93	94	91	96	93	146	280
**$Ding_{DT}$**	58	47	87	13	75	66	72	94	98	178	93	83	96	91	20	97	100	94	94	94	95	94	111	289
**$Jiang_{NB}$**	94	68	94	99	98	90	100	100	100	21	72	65	84	78	21	100	100	85	86	81	85	87	274	295
**Gao_{NB}**	90	55	90	94	96	85	100	100	100	59	77	71	85	82	41	100	100	89	90	86	89	91	239	298
**$Gudys_{DT}$**	82	54	85	17	83	71	26	91	100	160	93	82	93	90	90	96	100	92	93	90	93	93	142	302
**$Jiang_{DT}$**	89	51	85	33	92	67	37	97	97	142	92	76	91	89	69	100	100	93	94	90	93	95	160	302
**$Bentwich_{DT}$**	73	49	87	13	76	69	22	100	100	172	92	82	94	89	88	98	100	93	93	93	91	93	131	303
**$Chen_{DT}$**	60	52	85	34	81	68	44	98	100	158	93	78	93	89	61	92	100	93	94	91	94	94	150	308
**ConsensusRule**	94	65	94	95	98	86	87	100	100	28	76	59	84	84	3	100	100	83	83	80	83	87	281	309
**Xu_{DT}**	78	64	83	97	89	75	45	94	100	112	93	75	91	83	58	100	100	90	91	89	92	91	199	311
**$Batuwita_{DT}$**	76	53	85	21	88	71	30	100	100	145	90	78	93	88	91	98	100	91	93	89	93	90	167	312
**$Lopes_{DT}$**	57	51	84	17	75	70	27	90	97	197	90	80	91	89	87	96	100	95	96	92	97	95	133	330
**Gao_{DT}**	73	63	84	90	87	70	48	94	100	137	93	81	92	84	58	100	100	88	88	86	89	88	195	332
**$Chen_{NB}$**	73	54	88	90	89	79	9	100	100	124	78	76	89	86	99	100	100	88	89	84	90	92	208	332
**$Ritchie_{NB}$**	91	69	91	98	95	90	100	100	100	42	70	63	80	75	28	100	100	83	84	80	83	85	292	334
**Xu_{NB}**	92	81	92	100	96	92	100	100	100	26	71	62	81	66	2	100	100	81	81	78	79	85	308	334
**Xue_{NB}**	92	81	92	100	96	92	100	100	100	32	65	56	75	66	2	100	100	81	81	78	79	85	313	345
**$Burgt_{NB}$**	89	56	81	88	98	72	79	100	100	94	80	73	90	82	88	100	100	66	68	64	69	64	276	370
**$Ritchie_{DT}$**	76	49	83	66	83	72	67	94	100	150	92	76	91	86	36	98	100	84	85	82	86	85	236	386
**$Burgt_{DT}$**	77	51	85	61	79	73	49	92	99	147	90	76	90	85	57	93	99	86	88	83	86	86	243	390
**Xue_{DT}**	73	63	78	95	81	73	47	82	98	150	92	71	83	79	71	95	100	87	89	84	87	87	244	394

Supplementary Table 4: Model performance of all models versus all datasets. Sorted by total rank (lower is better). The values presented are true prediction rates achieved for each model and dataset.

Study	Learner	Recall	Precision	SN	SP	Fm	ACC	kappa	Yi	p+	p-
**Batuwita**	**DT**	0.88	0.87	0.88	0.88	0.86	0.86	0.72	0.76	7.45	0.28
**Batuwita**	**NB**	0.91	0.90	0.91	0.91	0.88	0.88	0.75	0.83	10.44	0.21
**Batuwita**	**SVM**	0.85	0.83	0.85	0.85	0.81	0.81	0.62	0.70	5.78	0.35
**Bentwich**	**DT**	0.89	0.88	0.89	0.89	0.87	0.87	0.74	0.77	7.85	0.24
**Bentwich**	**NB**	0.97	0.95	0.97	0.97	0.86	0.84	0.69	0.93	29.50	0.12
**Bentwich**	**SVM**	0.94	0.91	0.94	0.94	0.83	0.82	0.63	0.87	14.69	0.24
**Burgt**	**DT**	0.88	0.87	0.88	0.88	0.85	0.85	0.69	0.76	7.45	0.30
**Burgt**	**NB**	0.91	0.90	0.91	0.91	0.87	0.87	0.74	0.82	9.98	0.21
**Burgt**	**SVM**	0.90	0.87	0.90	0.90	0.82	0.81	0.61	0.81	9.36	0.25
**Chen**	**DT**	0.89	0.88	0.89	0.89	0.86	0.86	0.72	0.78	8.15	0.25
**Chen**	**NB**	0.93	0.92	0.93	0.93	0.86	0.86	0.71	0.87	14.25	0.21
**Chen**	**SVM**	0.94	0.94	0.94	0.94	0.91	0.91	0.83	0.89	16.71	0.16
**Ding**	**DT**	0.90	0.89	0.90	0.90	0.88	0.88	0.76	0.80	8.98	0.23
**Ding**	**NB**	0.92	0.91	0.92	0.92	0.89	0.89	0.78	0.84	11.48	0.18
**Ding**	**SVM**	0.91	0.90	0.91	0.91	0.88	0.88	0.77	0.83	10.44	0.21
**Gao**	**DT**	0.87	0.86	0.87	0.87	0.85	0.85	0.69	0.73	6.42	0.29
**Gao**	**NB**	0.95	0.93	0.95	0.95	0.87	0.86	0.72	0.89	17.30	0.20
**Gao**	**SVM**	0.91	0.90	0.91	0.91	0.87	0.86	0.73	0.83	10.44	0.25
**Gudys**	**DT**	0.88	0.87	0.88	0.88	0.87	0.87	0.73	0.75	7.07	0.25
**Gudys**	**NB**	0.92	0.91	0.92	0.92	0.88	0.87	0.75	0.84	11.77	0.20
**Gudys**	**SVM**	0.87	0.84	0.87	0.87	0.82	0.81	0.63	0.74	6.84	0.31
**Jiang**	**DT**	0.89	0.88	0.89	0.89	0.86	0.86	0.72	0.77	7.71	0.25
**Jiang**	**NB**	0.97	0.96	0.97	0.97	0.86	0.85	0.70	0.94	31.29	0.10
**Jiang**	**SVM**	0.87	0.86	0.87	0.87	0.84	0.84	0.68	0.73	6.52	0.29
**Lopes**	**DT**	0.87	0.87	0.87	0.87	0.85	0.85	0.70	0.75	6.96	0.26
**Lopes**	**NB**	0.90	0.89	0.90	0.90	0.86	0.86	0.72	0.80	8.80	0.22
**Lopes**	**SVM**	0.86	0.82	0.86	0.86	0.78	0.76	0.52	0.72	6.13	0.32
**Ng**	**DT**	0.95	0.94	0.95	0.95	0.90	0.90	0.80	0.89	17.93	0.19
**Ng**	**NB**	0.90	0.89	0.90	0.90	0.86	0.86	0.72	0.80	8.80	0.24
**Ng**	**SVM**	0.94	0.93	0.94	0.94	0.90	0.89	0.79	0.88	15.15	0.18
**Ritchie**	**DT**	0.85	0.84	0.85	0.85	0.83	0.83	0.65	0.69	5.54	0.32
**Ritchie**	**NB**	0.95	0.93	0.95	0.95	0.85	0.84	0.67	0.91	20.96	0.18
**Ritchie**	**SVM**	0.89	0.87	0.89	0.89	0.85	0.85	0.70	0.78	8.15	0.27
**Xu**	**DT**	0.86	0.85	0.86	0.86	0.84	0.84	0.68	0.71	5.95	0.32
**Xu**	**NB**	0.95	0.93	0.95	0.95	0.85	0.84	0.68	0.90	19.33	0.19
**Xu**	**SVM**	0.94	0.93	0.94	0.94	0.87	0.86	0.71	0.88	16.16	0.17
**Xue**	**DT**	0.81	0.79	0.81	0.81	0.78	0.78	0.56	0.62	4.28	0.43
**Xue**	**NB**	0.96	0.95	0.96	0.96	0.83	0.81	0.62	0.93	26.45	0.16

Supplementary Table 5: Model scores. Different measures calculated for each learning model produced. Here only the rows with maximum accuracy for each study/learner pair for the 1000 iterations are shown. The complete table is too large to reproduce here and is available for download on our webpage: http://jlab.iyte.edu.tr/software/izmir. Other than Youden's index, p+ and p- values, all of the measures are based on default KNIME settings. Performance measures calculated for all model testing were sensi-

tivity (SN), specificity (SP), recall, precision, F-measure (Fm), accuracy (ACC), Cohen's kappa (kappa), and Youdens's index (Yi) as well as p+ and p-.

	**Number of Hairpins**	
**Prediction score interval**	**dme mod-el**	**hsa model**
< 0.5	208,905	231,317
>= 0.5 & < 0.75	211,963	240,179
>= 0.75 & < 0.80	39,392	40,258
>= 0.80 & < 0.85	53,333	29,893
>= 0.85 & < 0.90	28,423	20,581
>= 0.90 & < 0.95	21,501	11,461
>= 0.95 & < 0.99	18,366	7,609
>= 0.99	0	585
Total	581,883	581,883

Supplementary Table 6: Hairpin number distribution based on prediction score interval. Prediction score cutoff versus number of hairpins selected from the pool of about 600,000 hairpin-like structures in the 2L chromosome of *Drosophila melanogaster*.
